# Influence of polishing methods on the physical properties of temporary restorations

**DOI:** 10.1007/s10266-025-01191-5

**Published:** 2025-09-15

**Authors:** Fabio Antonio Piola Rizzante, Zheng Qian, Pedro Henrique Magão, Italo Silva, Guilherme Faria Moura, Gustavo Mendonça

**Affiliations:** 1 Department of Reconstructive and Rehabilitation Sciences, James B. Edwards College of Dental Medicine, Medical University of South Carolina, Charleston, SC USA; 2 School of Dental Medicine, Case Western Reserve University, Cleveland, OH USA; 3 Department of Macromolecular Engineering, Case Western Reserve University, Cleveland, OH USA; 4 Department of General Practice, School of Dentistry, Virginia Commonwealth University Richmond, Richmond, VA USA

**Keywords:** Surface roughness, Surface properties, Polishing, Polymer, 3D printed resins

## Abstract

Although surface polishing of temporary restorations is a crucial clinical step, there is a lack of information about the effect of different polishing protocols on the surface properties of 3D printed resins for temporary restorations. This study had as goal to evaluate the effect of different polishing methods on the surface roughness, contact angle, and color stability of temporary restorations. One bis-acrylic resin (Integrity/IT) and 3 resins for 3D printing (Denture teeth, Formlabs/DT, Crown and Bridge MFH, Nextdent/CB, and Cosmos Temp, Yller/CT) were polished following 5 protocols (No polishing/Control, Jiffy Cup/JC, Jiffy Brush/JB, Sof-lex/SL, and Astropol/AS) and tested for surface roughness using a profilometer and for color stability using a spectrophotometer (after immersion in instant coffee for up to 7 days). Data were analyzed using 2-way ANOVA (surface roughness and contact angle) and 3-way repeated measurements ANOVA (color stability), all followed by Tukey test. Overall results of surface roughness showed CT > DT = CB = IT, and Control > JB > AS = SL = JC. The highest surface roughness was observed for IT control group, while the lowest values were observed for CB polished with SL. All groups showed lower surface roughness after being polished with multi-step polishing systems except for CB polished using AS, which showed similar values to CB polished with JB. Overall results of contact angle showed IT > CT > DT > CB, and JC > Control > AS > JB = SL. Highest contact angle was observed for CT control and all resins polished using JC, while the lowest values were observed for CB control group and CT polished using AS. Control groups for IT and CT were more hydrophobic than their respective polished groups, while CB and DT resins were more hydrophobic when polished compared to their respective control groups. Overall results of color variation showed CT > IT = CB > DT, and Control > JB > JC = SL = AS. All tested resins showed color variation higher than the 3.3 clinical threshold when immersed in coffee for 7 days, except DT and CT polished using JC. Different polishing methods can influence the physical properties of different resins used for temporary restorations. All materials showed better results when polished, especially when a multi-step polishing system was used.

## Introduction

Temporary restorations serve as an important restorative step to maintain teeth position, pulpal and periodontal health, occlusal stability, and to facilitate a better communication of the planned outcomes with patients and laboratory technicians [1–3]. A temporary restoration should be easy to fabricate and repair, have adequate mechanical properties to withstand physical–mechanical challenges while maintaining appropriate contour, present low surface roughness to prevent biofilm formation and changes in color, and adequate color stability, especially for esthetic cases and/or when temporaries are going to be used for a longer period of time [4–8].

Several materials are available for temporary restorations, including unfilled and filled resins, which can be obtained through conventional, digital, or combined workflows [1, 2, 4, 9]. Within this context, it is important for a temporary material to be fully integrated with the clinical workflow and with CAD/CAM technologies. Fast prototyping/3D printing is gaining popularity in clinics, offering an efficient clinical workflow with lower-cost equipment (3D printers), reducing waste, and providing similar adaptation to milling/subtractive manufacturing [3, 10–14]. As compared to fabrication of 3D printed temporary restorations using a digital workflow, fabrication of traditional temporary restorations using materials, such as acrylic and bisacrylic resins, requires more steps, such as obtaining a wax-up model and matrix or template, which increases clinical time and costs [2, 4].

Regardless of the restorative material choice, their surface should be optimally polished as a rough surface is associated with higher staining, biofilm accumulation, patient discomfort, tissue inflammation, and secondary decay. A commonly accepted threshold for surface roughness (Ra value) is 0.2 µm [2, 15], and higher values may result in tissue inflammation and jeopardize the final restoration [2, 16–18].

To achieve a polished surface, manufacturers of 3D printed materials often recommend surface glazing with different surface sealants or coating agents, as well as sequences of polishers. It is well documented in the literature that surface sealants are usually degraded during clinical use [19, 20], therefore, mechanical polishing is preferred. Within this context, literature on polishing systems for resin composites shows multi-step polishing systems usually result in better surface characteristics when compared with single-step polishing [3, 21].

Although finishing and polishing of resin composite restorations is well documented in the literature and play a major role in the material longevity and in the color stability of restorations [15, 21], there is a lack of information about surface properties of 3D printed resins used for temporary restorations.

Therefore, the objective of this study was to assess the effects of different polishing protocols on the surface roughness, surface hydrophilicity, and color stability of different 3D printed resins used for temporary restorations.

The null hypothesis tested was:

- there would be no difference in surface roughness considering the different polishing methods and resins;
- there would be no difference in contact angle considering the different polishing methods and resins;
- there would be no difference in color stability considering the different polishing methods and resins;

## Material and methods

This study assessed different temporary resins in 4 levels and different polishing methods in 5 levels (Table 1) having as response variables the surface roughness (evaluated with a laser scanning microscope), contact angle (evaluated with a video contact angle analysis system), and color stability after immersion in instant coffee for up to 7 days (evaluated with a spectrophotometer).

**Table 1:** List of materials used in the present study

Material	Manufacturer	Material type
Integrity/IT	Dentsply Sirona	Bisacrylic resin
Denture teeth/DT	Formlabs	3D printed resin
Crown and Bridge MFH/CB	Nextdent	3D printed resin
Cosmos Temp/CT	Yller	3D printed resin
No polishing/Control	N/A	Polishing Method
Jiffy Brush/JB	Ultradent	1-step polishing method
Jiffy Cup/JC	Ultradent	3-step polishing method
Sof-lex/SL	3 M ESPE	3-step polishing method
Astropol/AS	Ivoclar Vivadent	3-step polishing method

Fifty specimens were made for each tested resin and randomly divided into 5 subgroups according to the polishing method (Table 1). For the bis-acrylic resin, IT was injected in a 10 mm diameter × 2 mm height PVS mold, covered with a mylar strip and glass slide to standardize the surface and dimensions, and was left in the mold for 15 min for complete polymerization. The 3D printed resins (DT, CB, and CT) were designed using an open CAD software (Meshmixer v.3.5.474; Autodesk), exported as stereolithography files (.STL), and printed using a stereolithography-based (SLA) 3D printer (Form 2; Formlabs) using preform software (v.3.1.2). All samples were printed with their circular faces 90º to the build plate as it is reported to result in the best mechanical properties and require less supports and post-processing (such as trimming and polishing) [5, 10]. The supports were set to a density of 1, point size of 600 μm, and layer thickness of 100 μm, and the resin parameter set to “denture teeth” [10]. The 3D printed samples were detached from the build platform, washed in isopropyl alcohol at 99.5% using a Formwash (Formlabs) for 20 min, followed by post-polymerization using the Formcure (Formlabs) for 30 min at 80 ºC, immersed in glycerin.

Samples were stored in a dark, dry storage at 37 ºC for 24 h, followed by polishing using the different methods listed on Table 1. With the exception of the control group, all samples were polished by a previously calibrated operator. Each specimen was polished for 20 s with each polisher using a slow-speed handpiece at 7000 revolutions per minute (RPM), following the sequence from the coarsest to the smoothest when available. Polishing was performed keeping the same brushing motion in one axis of the sample for 10 s, followed by polishing in a perpendicular direction for the remaining 10 s. The polishers were changed every 3 specimens and polished resin specimens were immersed in an ultrasonic bath for 5 min to remove debris. Each specimen was used for all tests (surface roughness, contact angle, and color stability).

Roughness was tested using a laser confocal microscope (LEXT OLS 4100; Olympus Corporation), with 0.1 μm X–Y resolution and 0.01 μm Z resolution. Specimens were positioned under the 20 × magnification lens and 4 different areas (646 μm × 644 μm) separated by at least 1 mm were analyzed using the built-in measurement tools. The average Arithmetical Mean Height (Sa) values were recorded for each specimen.

For the contact angle test, 10 μL of distilled water was dropped over the specimen surface and the angle formed on both left and right sides of the water drop was analyzed using the built-in software of a Video Contact Angle Analysis System (VCA 2500XE; AST Products Inc.). The experiment was repeated 3 times for each specimen, at random areas of the specimen’s surface, and the average of all readings was considered for analysis.

The staining test was performed after polishing (baseline), and after 1, 3, and 7 days of immersion in instant coffee. Five grams of instant coffee powder (Traditional Nescafé; Nestlé) were added to 200 mL deionized water pre-heated to 100 ºC. The solution was allowed to cool to 37 ºC, and 10 mL was poured into each specimen flask. The solution was replaced every 24 h, and the specimens were stored in an incubator at 37 ºC for the entire length of the experiment.

A CIE-lab based colorimeter was used (Easyshade; Vita Zahnfabrik). The colorimeter was calibrated before each reading time point. Each specimen was rinsed under deionized water, dried with absorbent paper, and placed over a white sheet (to standardize the background). Three readings were performed perpendicular to each specimen surface until homogeneity of results was observed. The average of the 3 readings was considered as the final color of each specimen [1, 9] and the color change was calculated based on the following formula:

$$ \Delta {\mathrm{E}} = \left( { \, \Delta {\mathrm{L}}*} \right)^{{2}} + \, \left( {\Delta {\mathrm{a}}*} \right)^{{2}} + \, \left( {\Delta {\mathrm{b}}*} \right)^{{2}} $$

In which ΔL*, Δa*, and Δb* correspond to the color differences observed between the baseline and the subsequent measurements.

Data was assessed for normality using the Kolmogorov–Smirnov test and analyzed using 2-way ANOVA (surface roughness and contact angle) and 3-way repeated measurements ANOVA (color stability), all followed by the Tukey test. All statistical analyses were performed adopting a 5% significance level.

## Results

**Fig. 1:**
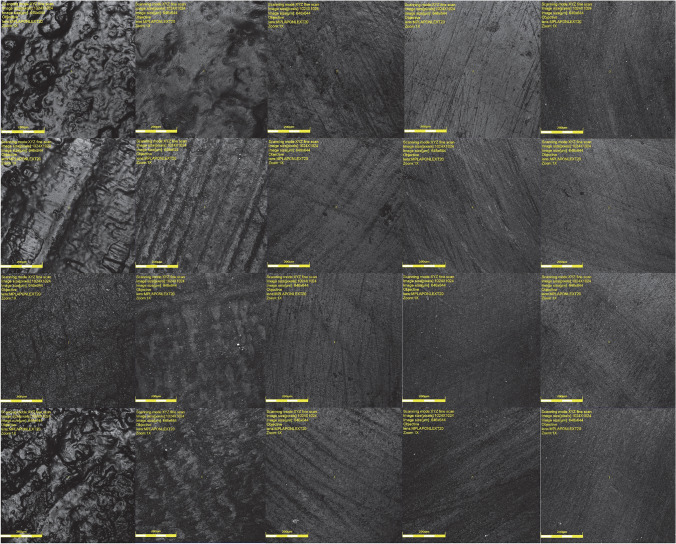
Surface characteristics under laser confocal microscope. From left to right: Control, JB, JC, SL, AS. From top to bottom: Integrity, Denture teeth, Crown and Bridge MFH, and Cosmos Temp

Results of surface roughness can be observed in Table 2 and example images can be observed in Fig. 1. Resin, polishing method, and interaction between both factors were statistically significant (*P* < 0.001 for all factors). Overall results showed CT > DT = CB = IT, and Control > JB > AS = SL = JC. The highest surface roughness was observed for IT control group, while the lowest values were observed for CB polished with SL. All groups showed lower surface roughness after being polished with multi-step polishing systems except for CB polished using AS, which showed similar values to CB polished with JB.

**Table 2:** Roughness (std dev) values in Sa for tested groups

	Integrity	Denture teeth	Crown and bridge	Cosmos temp
Control	1.13 (0.15)Aa	1.22 (0.27)Aa	1.12 (0.08)Aa	2.13 (0.51)Ab
Jiffy brush	0.86 (0.16)Aa	0.6 (0.16)Cb	0.52 (0.1)Bb	0.84 (0.18)Ca
Jiffy cups	0.35 (0.07)Ba	0.3 (0.03)Da	0.27 (0.03)Ca	0.53 (0.05)Db
Sof-lex	0.26 (0.07)Ba	0.35 (0.07)Da	0.24 (0.04)Ca	0.41 (0.12)BDb
Astropol	0.28 (0.06)Ba	0.21 (0.04)Ba	0.52 (0.1)Bb	0.28 (0.04)Ba

Capital letters—difference between polishing methods considering the same resins

Lowercase letters—difference between resin groups considering the same polishing methods

Results of Contact Angle can be observed in Table 3. Resin, polishing method, and interaction between both factors were statistically significant (*P* < 0.001 for all factors). Overall results showed IT > CT > DT > CB, and JC > Control > AS > JB = SL. The highest contact angle was observed for CT control, and all resins polished using JC, while the lowest values were observed for CB control group and CT polished using AS. Control groups for IT and CT were more hydrophobic than their respective polished groups, while CB and DT resins were more hydrophobic when polished compared to their respective control groups.

**Table 3:** Contact angle (std dev) values for tested groups

	Integrity	Denture teeth	Crown and bridge	Cosmos temp
Control	90.74 (6.26)ABa	54 (6.67)Ab	36.65 (3.16)Ac	104.29 (2.72)Aa
Jiffy brush	60.55 (2.14)Ca	63.38 (5.8)ABa	45.2 (4.48)ACb	66.51 (4.22)Ca
Jiffy cups	98.01 (2.13)Aa	94.89 (2.22)Ca	99.7 (1.91)Da	96.89 (2.04)Aa
Astropol	80.38 (7.38)Ba	74.66 (7.08)Ba	74.08 (0.87)Ba	40.92 (6.79)Bb
Sof-lex	54.83 (4.93)Ca	63.28 (4.61)ABa	57.54 (3.67)Ca	61.15 (8.74)Ca

Capital letters—difference between polishing methods considering the same resins

Lowercase letters—difference between resin groups considering the same polishing methods

**Fig. 2:**
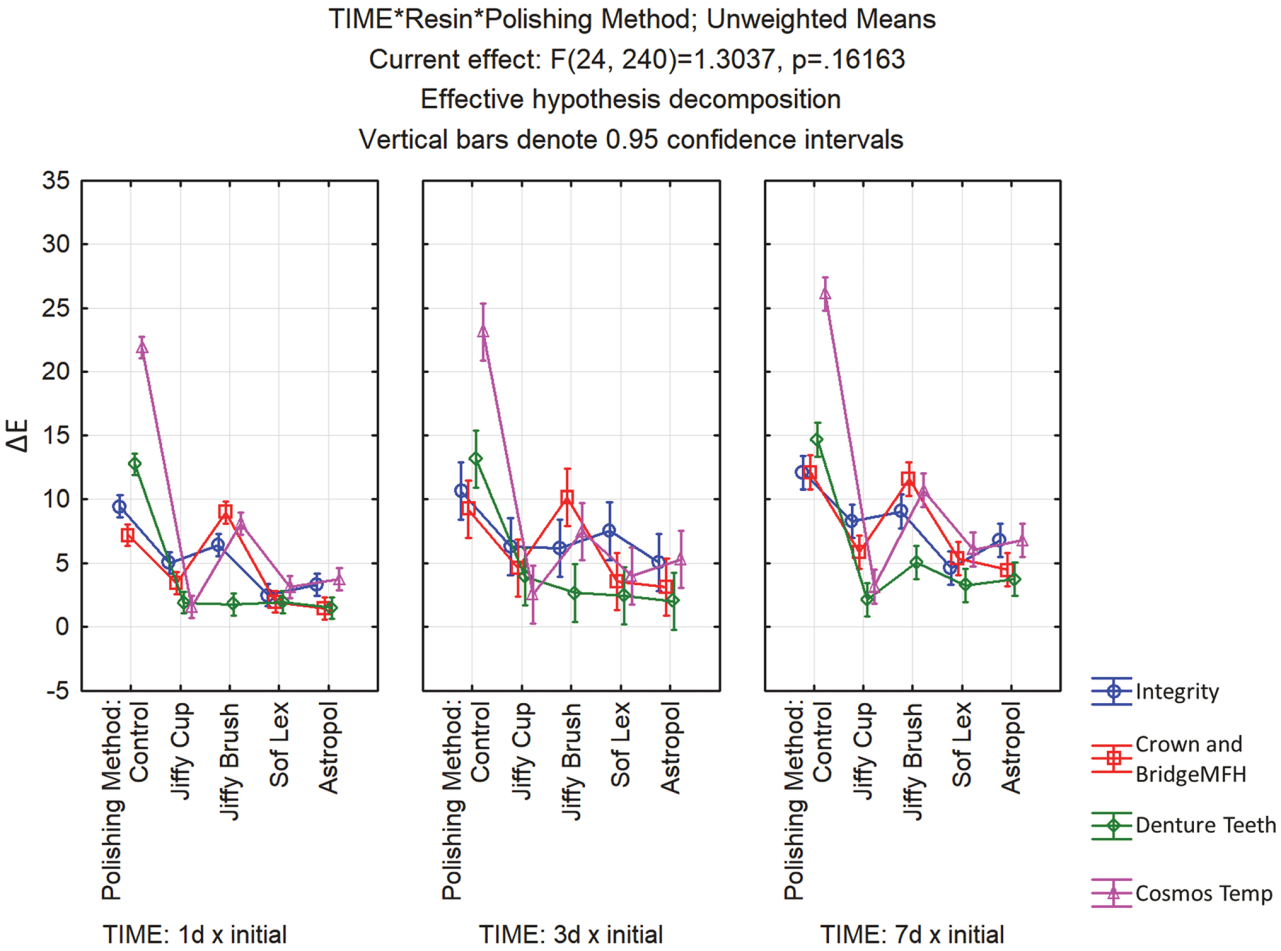
Results for color variation (delta E) for tested groups

Results of color variation can be observed in Fig. 2. Statistically significant differences were observed for resin, polishing method, time, and interaction between resin and polishing method (*P* < 0.001 for all factors). Overall results of color variation showed CT > IT = CB > DT, and Control > JB > JC = SL = AS. All tested resins showed color variation higher than the 3.3 clinical threshold after immersion in coffee for 7 days, except DT and CT polished using JC.

## Discussion

All null hypotheses were rejected as all evaluated properties (surface roughness, contact angle, and color stability) were different based on the tested resins and polishing methods.

Surface roughness of a restorative material is important as it correlates with color stability and biofilm accumulation, which may result in unaesthetic results if the temporaries are used for a longer period of time, as well as in tissue inflammation, potentially compromising the clinical steps necessary to achieve a long-lasting final restoration [2, 3, 16, 17].

Although the comparison between surface roughness of 3D printed and conventional temporary materials is controversial and largely dependent on composition of tested resin and printing orientation [5, 6], this study results are in agreement with the literature [12], and offers some understanding about the importance of adequate polishing protocol on the final properties of 3D printed parts.

In this study, surface roughness was influenced by resin, polishing method, and association of both factors (*P* < 0.001). Overall results showed CT resin presented higher surface roughness than all tested materials. Considering the polishing methods, all polished resins showed decreased surface roughness when compared with their respective control groups, except IT polished with JB. Comparing the different polishers, JB usually resulted in higher roughness when compared with other polishing methods, except when compared with AS used to polish CB resin.

This tendency is explained by the number of clinical steps involved in the different polishing protocols. While JB is a single-step polisher, all other polishing systems tested in this study are 3-step polishers. Such results are in agreement with recent literature showing that the most effective polishing systems are those using a decreasing size of abrasive particles, or multi-step [3, 21].

Moreover, part of the results may be explained by the adoption of a surface roughness based on an entire area (Sa) instead of based on a line (Ra), which provides a more comprehensive overview of the surface characteristics.

Interestingly, when comparing the different polishers used for each resin, it shows that IT and DT, which are unfilled resins, were similarly polished with all 3-step polishers, which can be explained by a softer resin surface, which is easier to polish [3]. For CB and CT, which have some filler content, the same tendency was observed, except that for CB, JC and SL resulted in better polishing than AS, and for CT, AS resulted in better polishing than JC and SL.

This can be explained by the interaction between polishers and different compositions of the resins. Sof-lex discs and Jiffy-cups are based on silicon-oxide particles, while Astropol is based on diamond-impregnated silicon. While both aluminum oxide and diamond-impregnated polishers are considered equally effective for resin composites [3], it can be speculated that filler contents present in some 3D printed materials may be easier to polish with a certain polisher. This is corroborated by the fact that Jiffy cups and Sof-lex discs presented similar results, which were significantly better or worse when compared to Astropol. Nevertheless, very little information is provided by the manufacturer, and further research is encouraged to better understand the materials’ organic and inorganic composition.

All materials presented similar roughness considering their control groups, except for CT, and lower roughness after the different polishing protocols, which evidence the effectiveness of the study protocol. Figure 1 shows some examples of the interaction between different resins and polishers. It is very clear the amorphous surface characterized by non-polymerized resin in the control group, with some improvement after 1-step polisher (JB). Multi-step polishers showed a complete removal of the non-polymerized resins and polished surfaces characterized by some remaining scratches, probably due to the polisher grit size. It can be speculated that a longer polishing time may have further improved the surface.

Although Sa and Ra cannot be directly compared, it can be inferred that the 0.2 μm Ra threshold for plaque accumulation [3, 17] would be desirable as a value for Sa, since it would be even harder to obtain as it is based on an entire area rather than on a line. That being said, all materials were above that threshold, which is in agreement with recent literature about 3D printed parts [2, 7, 9, 19].

Contact angle can be correlated with the hydrophilicity of a material, which can be an indicator of the color stability. Contact angle can also indicate the surface energy, which can be an indicator of surface roughness [8]. In this study, contact angle is influenced by both material and polishing method. The difference in the control groups indicates the different compositions of the tested materials.

Interestingly, while for IT and CT, polishing methods either maintained or reduced the contact angle when compared with the respective control groups, for DT and CB, all polishing methods resulted in an increased contact angle.

The results for DT and CB may indicate the material surface was not fully polymerized and a sub-optimally polymerized resin was removed during the polishing protocol, resulting in increased contact angle. For both IT and CT, only samples polished using JC (and AS for IT) showed contact angles similar to the respective control groups.

Results of color variation can be observed in Fig. 2. Statistically significant differences were observed for resin, polishing method, time, and interaction between resin and polishing method (*P* < 0.001 for all factors). Overall results of color variation show CT > IT = CB > DT, and Control > JB > JC = SL = AS.

Overall, color stability tests also showed a tendency for better color stability when multi-step polishing protocols were used, and the worst color alteration when resins were not polished: Control > JB > JC = SL = AS. Despite the differences, all tested resins showed color variation higher than the 3.3 clinical threshold after immersion in coffee for 7 days, except DT and CT when polished using JC. Interestingly, the 2 materials showed the highest contact angle when polished with JC, although not the lowest surface roughness, which may indicate surface hydrophilicity may be more relevant for color stability than surface roughness, provided a certain level of polishing is achieved. The lack of correlation between surface roughness and color stability, as well as the overall lack of color stability for 3D printed resins is in agreement with other studies [2, 8, 13, 14, 22, 23].

Despite many advantages of the digital workflow, the lack of color stability for 3D printed parts is an important issue widely reported in the literature. Color alterations are attributed to water absorption and hydrolysis, due to the association between low filler content and a methacrylate-based matrix [2, 4, 7, 9].

One could question about the advantages of mechanical surface polishing versus use of surface coatings since the latter is technically easier and faster. Nevertheless, it has been reported that such surface coating deteriorates and results in higher color changes when compared with adequately polished surfaces [13, 18, 19].

This study focused on assessing the effects of different polishing protocols on the surface properties of materials for temporary restorations. It is noteworthy that 3 materials for 3D printing were tested (2 filled and 1 unfilled), and a bis-acrylic resin was used as a reference for the conventional techniques for the manufacturing of temporary restorations. For the 3D printed materials, only one post-polymerization protocol was used to standardize the groups, and different workflows may influence the results. Future studies should assess the influence of the polishing protocols on resins indicated for definitive restorations and those claiming to be 3D printed ceramics. Moreover, other polishing protocols should be studied, as well as the interaction between post-polymerization protocols and polishing methods.

All specimens were printed with a 90° angle as it has been reported to result in the best mechanical properties and require less support and post-processing (such as trimming and polishing), preventing the creation of defects on the surface and/or a decrease in mechanical properties [5, 10]. Future studies should focus on the effects of different equipment and parameters on the final properties of 3D printed parts to better explain the controversial results in the literature.

One may ask about temporary blocks for CAD-CAM and, although they are also integrated with the digital workflow, the equipment and material are far more expensive than some 3D printers and 3D resins available in the market. In addition, there are current limitations related to shaping complex details and manufacturing speed when long span temporaries are needed [3].

Coffee has a high staining potential because particles are absorbed and adsorbed on the resin composite surfaces and into the matrix [20]. In the present study, 7 days of coffee immersion was chosen because it has been reported that the greatest proportional amount of discoloration occurs within the first week, thus allowing the prediction of a material’s long-term color stability [20].

## Conclusion

Different polishing methods can influence the physical properties of different resins used for temporary restorations. All materials showed better results when polished, especially when a multi-step polishing system was used. Three-dimensionally printed provisional materials showed similar or better surface properties compared to bis-acrylic resins after polishing.
